# Dataset on risk factors for seroconversion against *Mycobacterium avium* subspecies *paratuberculosis* in dairy cows

**DOI:** 10.1016/j.dib.2023.109671

**Published:** 2023-10-13

**Authors:** E. Nunney, M. Crotta, K. Bond, S. van Winden, M. Green, J. Guitian

**Affiliations:** a Veterinary Epidemiology, Economics and Public Health Group, OIE Collaborating Centre in Risk Analysis and Modelling, Department of Pathobiology and Population Sciences, The Royal Veterinary College, Hawkshead Lane, North Mymms, Hatfield, Herts, AL9 7TA, UK; b National Milk Records Group. Fox Talbot House, Greenways Business Park, Bellinger Close, Chippenham, Wiltshire, SN15 1BN, UK; c The School of Veterinary Medicine and Science, University of Nottingham, Sutton Warwickshire, CV8 2TL Bonington Campus, Sutton Bonington, Leicestershire, LE12 5RD, UK

**Keywords:** Johne's disease, Infection, calves, MAP, Milk ELISA

## Abstract

Johne's disease (JD) is a chronic wasting disease caused by *Mycobacterium avium* subspecies *paratuberculosis* (MAP). MAP is responsible for large economic losses for the dairy sector and has been linked to human disease. Susceptibly to MAP is mainly limited to young animals and diagnostic tests are poor at detecting MAP in early stages of infection. Therefore, ascertaining the contribution of the dam to the risk of calf infection and the relative role of the different infection routes is important to inform disease control measures. This data article presents MAP exposures at time of calving on a cohort of 439 calves born between 2012 and 2013 from 6 UK dairy herds. Each calf participated in routine quarterly MAP milk ELISA testing using the IDEXX Porquire ELISA. Each animal was followed until testing MAP positive, being culled or end of follow up (January 2023). The dataset includes risk factors associated with transmission via colostrum route (MAP status of cow giving colostrum); transmission via the dam (MAP status of the dam) and transmission via fecal oral route (whether at birth the calf spent a long time in a dirty yard). Ascertainment of exposure to risk factors involved video recording and self-capture data forms from time of calving in the maternity area of the farms until calf left the area. The dataset provides a unique opportunity to examine MAP infection and its relationship with different exposures at time of birth, where cows were followed up during their entire lives.

Specifications Table

**Table:** 

Subject	Veterinary Epidemiology
Specific subject area	Epidemiology of *Mycobacterium avium* subsp. *paratuberculosis* (MAP) infection in dairy cows
Data format	Raw
Type of data	Table
Data collection	Data were collected for 439 cows born 2012-2013 from 6 UK dairy herds to assess potential risk factors for MAP infection at time of calving, using: i) information gathered through video recordings of the calving area, ii) self-capture data forms filled in by farmer and iii) MAP status of other cows from the same herd. The MAP status of each animal in a herd was determined through routine quarterly milk testing for presence of antibodies against MAP using the IDEXX Porquier ELISA, with test results obtained from milk recording companies. Data collection of test results ceased when an animal tested positive, was culled or at the end of study (January 2023).
Data source location	Data stored at the Royal Veterinary College, London, UK.
Data accessibility	Repository name: Dataset on risk factors for seroconversion against Mycobacterium avium subspecies paratuberculosis in dairy cows. Data identification number: 10.17632/8cwj8h5h65.1 Direct URL to data: https://data.mendeley.com/datasets/8cwj8h5h65/3
Related research article	E. Nunney, M. Crotta, S. van Winden, K. Bond, M. Green and J. Guitian, Unravelling transmission of Mycobacterium avium subspecies paratuberculosis to dairy calves: results of a lifelong longitudinal study, Prev. Vet. Med. (2023). https://doi.org/10.1016/j.prevetmed.2023.106022

## 1 Value of the Data

• The data allow exploration of associations between cow exposures and MAP infection, given that infection takes place soon after birth but usually remains undetectable for years, a dataset that assesses disease status until the end of the life of the animal is particularly valuable.
• There are important knowledge gaps regarding MAP infection of dairy cows, including the relative role of the different infection routes, which are important to inform control measures. These data combined with other data sources can generate insights into the relative role of different infection routes.
• These data can be useful for researchers interested in the epidemiology of MAP infection and the dairy industry given the impact of MAP infection on health, production and welfare of dairy cows and for the public given concerns regarding the potential public health implications of human exposure to MAP.
• Researchers interested in the epidemiology of MAP infection in dairy herds with access to additional data may be able to combine these data with theirs. The data can also support studies on the dynamics of MAP infection within dairy herds.

## 2 Data Description

The dataset contains variables used to assess exposure of each individual cow to potential risk factors for MAP infection. The recorded variables were obtained by combining data obtained from three different sources:

(i) Examination of video footage obtained from cameras recording the calving areas of the 6 study farms
(ii) Self-capture data forms filled in by farmers at and around time of calving
(iii) Results from routine testing of milk samples for presence of antibodies against MAP from other cows from the same herds provided by milk recording companies.

Each row in the dataset represents data on each animal (439 cows from 6 different herds).

The data file (data.xlsx) and data dictionary (dict.pdf) are deposited at the Mendeley repository. Information about the columns in the dataset is presented in Table 1.

**Table 1:** Description of variables present in the dataset.

Column name	Definition	Source
cow_id	Unique ID for each animal	Self-capture data form
herd	Unique ID for each farm	Self-capture data form
dob	Date of birth of animal	Self-capture data form
start_date	Date of first MAP milk ELISA test recording	Milk recording data
end_date	Date of last MAP milk ELISA test recording. An animal's first positive test result would be marked as there last recording in the study.	Milk recording data
time_in_pen	Time spent in the maternity yard from calving till time calf left maternity area. Time recorded in minutes.	Video footage
cow_st	MAP status of animal between 2012 and 2023	Milk recording data
col_source1	Source of colostrum. Either Pooled / Dam / Other cow / Artificial.	Self-capture data form
col_source2	Source of colostrum from secondary source. Either Pooled / Dam / Other cow / Artificial.	Self-capture data form
yd_clean	Cleanliness of the maternity area at time of calving measured by Wisconsin scoring system.	Video footage
dam_st	MAP status of the dam, between 2012 and 2023.	Milk recording data
col_st1	MAP status of cow giving colostrum	Milk recording data
col_st2	MAP status of secondary cow giving colostrum	Milk recording data
col_st	MAP status of cow giving colostrum between 2012 and 2023, recorded as positive if either MAP status of primary cow or secondary cow giving colostrum were positive. Artificial colostrum assumed negative. Calves given pooled colostrum were treated as missing data, due to uncollected data from contributing cows.	Generated from col_st1, col_st2, col_source
lg_time_dirty	Long time in dirty yard was recorded as animals spending ≥ 439 minutes in the maternity yard and having a cleanliness score of ≥ 3.	Generated from yard_cl and time_in_pen
map_route	Animals categorized into two groups depending on route of MAP infection. Animals classified as having MAP infection via dam or colostrum if MAP status of dam and cow giving colostrum were both positive. Animals classified as having MAP infection not via dam or colostrum if MAP status of dam and cow giving colostrum were both negative.	Generated from dam_st and col_st

## 3 Experimental Design, Materials and Methods

The goal of the accompanying paper was to unravel the roles different transmission routes play on MAP infection of dairy calves [1]. To achieve this aim, a longitudinal study was conducted. Female calves born between September 2012 to August 2013 from 6 selected UK dairy herds were enrolled. Of the 600 calves enrolled (161 lost prior to first milk recording), 439 calves were followed up to examine if the outcome of interest (MAP positive test result) occurred during their lives.

Exposure measurements were obtained at time of calving. All calves were born in large multiple cow maternity pens/yards. Cameras were placed in calving areas of the maternity yard. The footage was examined by one researcher from the point of calving until removal of each calf from the calving yard. Yard cleanliness was measured from examining the video footage of the maternity area using a standardized scoring system [2]. The time spent in the yard was measured, using the video footage, from the time the calf was born until it was removed to the calf house.

A standardized paper-based form was filled in by the farmer after each calf was born on the farm, to record colostrum source (pooled, artificial, dam, other cow), dam ID and ID of cow giving colostrum. Forms were collected on a weekly basis by the researcher.

The variable time spent in dirty pen was created from the variable's cleanliness of the pen and time spent in pen. The median time spent in a pen from calves in the study (439 minutes) and a cleanliness score of 3 was used as the cut-off to group animals into long time spent in dirty yard versus not spending a long time in a dirty yard.

The outcome of interest in our study (MAP status of each of the 439 recruited cows) along with MAP status of the dam and of cows giving colostrum to recruited cows (when different from the dams) were determined from routine MAP testing, conducted as part of the Herdwise programme [3]. Milk samples were collected from all cows in the milking herd on a quarterly basis and tested for the presence of MAP antibodies using the IDEXX Porquier ELISA. The routine milk ELISA test results recorded by National Milk Recording (NMR) and Cattle Information Services (CIS) were requested for the study period (2012 to January 2023). The test results provided by the companies were given as sample-to-positive ratios (S/P). A cow was defined as MAP positive if the S/P was greater or equal to 0.3. Any positive test result between 2012 and 2023 led to the categorisation of the respective animal as positive for MAP in the dataset.

The variables start and end date corresponded to the first MAP milk ELISA test and the end test date for each individual animal. The end point for a MAP positive animal was determined as the date of its first positive test result.

Data from the different sources (MAP milk test results and exposure measurements at time of calving) were combined matching by cow ID and cleaned using statistical software R [4].

## 4 Limitations

MAP status of an animal relied on the use of an imperfect diagnostic test (IDEXX Porquier ELISA; sensitivity = 0.62, specificity = 0.99 [5]). The assessment of yard cleanliness depended on subjective judgment by the researcher. Missing data occur for some variables e.g., as a result of failures with camera equipment, video footage was unavailable to assess the time spent by animals in pens.

## Ethics Statement

The work does not involve human subjects, animal experiments, or any data collected from social media platforms.

Ethics permission for this study was granted by the Royal Veterinary College (URN 2012 1145).

## CRediT authorship contribution statement

**E. Nunney:** Writing – original draft, Data curation, Software. **M. Crotta:** Data curation, Software. **K. Bond:** Resources. **S. van Winden:** Conceptualization, Writing – review & editing. **M. Green:** Writing – review & editing, Supervision. **J. Guitian:** Conceptualization, Methodology, Writing – review & editing, Supervision, Funding acquisition.

## Data Availability

Dataset on risk factors for seroconversion against Mycobacterium avium subspecies paratuberculosis in dairy cows. (Original data) (Mendeley Data)
